# Mammalian ribosomal and chaperone protein RPS3A counteracts α-synuclein aggregation and toxicity in a yeast model system

**DOI:** 10.1042/BJ20130417

**Published:** 2013-10-10

**Authors:** Stijn De Graeve, Sarah Marinelli, Frank Stolz, Jelle Hendrix, Jurgen Vandamme, Yves Engelborghs, Patrick Van Dijck, Johan M. Thevelein

**Affiliations:** *Department of Molecular Microbiology, VIB, KU Leuven, Kasteelpark Arenberg 31, B-3001 Leuven-Heverlee, Flanders, Belgium; †Laboratory of Molecular Cell Biology, Institute of Botany and Microbiology, KU Leuven, Kasteelpark Arenberg 31, B-3001 Leuven-Heverlee, Flanders, Belgium; ‡Biochemistry, Molecular and Structural Biology section, KU Leuven, Celestijnenlaan 200G, B-3001 Leuven-Heverlee, Flanders, Belgium

**Keywords:** component of oligomeric Golgi complex 6 (COG6), Parkinson’s disease, ribosomal and chaperone protein S3A (RPS3A), α-synuclein, yeast screening, COG6, component of oligomeric Golgi complex 6, ER, endoplasmic reticulum, 5-FOA, 5-fluoro-orotic acid, Gapdh, glyceraldehyde-3-phosphate dehydrogenase, HBx, hepatitis B protein X, HRP, horseradish peroxidase, LB, Lewy body, MPTP, 1-methyl-4-phenyl-1,2,3,6-tetrahydropyridine, NA, numerical aperture, PD, Parkinson’s disease, Pgk1, phosphoglycerate kinase 1, PSMA2, proteasome subunit α type 2, Spir1CT, C-terminus of Spire homologue 1, RPS3A, ribosomal protein S3A, SC, synthetic complete, SD, synthetic dextrose, SNARE, soluble *N*-ethylmaleimide-sensitive fusion protein-attachment protein receptor, αSyn, α-synuclein, WT, wild-type, yeGFP, yeast-enhanced GFP, YPD, yeast extract/peptone/dextrose

## Abstract

Accumulation of aggregated forms of αSyn (α-synuclein) into Lewy bodies is a known hallmark associated with neuronal cell death in Parkinson's disease. When expressed in the yeast *Saccharomyces cerevisiae*, αSyn interacts with the plasma membrane, forms inclusions and causes a concentration-dependent growth defect. We have used a yeast mutant, *cog6*Δ, which is particularly sensitive to moderate αSyn expression, for screening a mouse brain-specific cDNA library in order to identify mammalian proteins that counteract αSyn toxicity. The mouse ribosomal and chaperone protein RPS3A was identified as a suppressor of αSyn [WT (wild-type) and A53T] toxicity in yeast. We demonstrated that the 50 N-terminal amino acids are essential for this function. The yeast homologues of RPS3A were not effective in suppressing the αSyn-induced growth defect, illustrating the potential of our screening system to identify modifiers that would be missed using yeast gene overexpression as the first screening step. Co-expression of mouse RPS3A delayed the formation of αSyn–GFP inclusions in the yeast cells. The results of the present study suggest that the recently identified extraribosomal chaperonin function of RPS3A also acts on the neurodegeneration-related protein αSyn and reveal a new avenue for identifying promising candidate mammalian proteins involved in αSyn functioning.

## Abstract 2

A yeast screening system was developed and successfully used to isolate a brain protein that counteracts the toxic effects of the main protein involved in Parkinson's disease.

## INTRODUCTION

Since the discovery that a point mutation (A53T) in *SNCA*, the gene encoding αSyn (α-synuclein), causes an autosomal dominant form of PD (Parkinson's disease) [1], there has been a strong interest in the physiological function of this protein and its connection with PD. αSyn is a highly conserved, vertebrate-specific, 140-amino-acid protein that consists of an amphipathic N-terminal domain, an internal hydrophobic region and an acidic C-terminal tail. Misfolded αSyn is the major component of the fibrillar cellular inclusions called LBs (Lewy bodies), the pathological hallmark of PD [2]. The abnormal accumulation of αSyn in cellular inclusions has been associated with an entire spectrum of neurodegenerative diseases, collectively termed synucleinopathies [3]. Additional point mutations (A30P and E46K) in αSyn and mutations that lead to increased αSyn expression have been linked to autosomal dominant PD (as reviewed in [4]). Although familial PD caused by αSyn mutations is very rare, PD characterized by αSyn-containing LBs accounts for most of the sporadic cases of PD. Membrane-associated intermediates of fibrillization, or protofibrils, have been found to permeabilize presynaptic vesicles [5], interfere with vesicle trafficking [6], delay ER (endoplasmic reticulum)-to-Golgi transport by antagonizing ER/Golgi SNAREs (soluble *N*-ethylmaleimide-sensitive fusion protein-attachment protein receptors) [7] and inhibit lysosomal function [8] and chaperone-mediated autophagy [9]. Cytosolic protofibrils are thought to inhibit proteasomal protein degradation [10]. In dopamine-producing neurons these events lead to cell death through pathways that involve oxidative stress, mitochondrial dysfunction, ER stress and accumulation of misfolded proteins (as reviewed in [11]).

In yeast cells, human WT (wild-type) and A53T αSyn associate with the plasma membrane after which they form cytoplasmic inclusions [12]. Many characteristics of αSyn observed in other models have been described in yeast (as reviewed in [13,14]). Lindquist and colleagues have identified several multi-copy suppressors of αSyn toxicity, including *YPT1* and *YPK9*, the yeast homologues of human *RAB1* and *PARK9* respectively [15,16]. Liang et al. [17] have identified several other yeast genes that, upon overexpression, counteract αSyn-induced toxicity. The gene products played roles in ubiquitin-dependent protein catabolism, protein biosynthesis, vesicle trafficking and the response to stress.

We have taken the use of yeast as a tool for αSyn studies a step further. We have used the *cog6*Δ (COG6 is component of oligomeric Golgi complex 6) yeast deletion strain, which is highly sensitive to moderate levels of αSyn expression, to screen a brain-specific cDNA library for mammalian cDNAs that counteract αSyn toxicity in yeast. We have isolated a cDNA clone encoding ribosomal and chaperone protein RPS3A (ribosomal and chaperone protein S3A) that suppresses yeast αSyn toxicity, and it does this also independently from the *cog6*Δ deletion. The *Saccharomyces cerevisiae* homologues of RPS3A, Rps1A and Rps1B, were not effective in suppressing αSyn toxicity and thus could not have been identified in a yeast overexpression screen, which illustrates the power of our mouse brain cDNA screening strategy. Because RPS3A also strongly reduced αSyn–GFP inclusion formation, we suggest that its recently described chaperonin function [18] is involved in preventing αSyn from folding into a harmful conformation, possibly by stabilizing the recently described helically folded form [19].

## EXPERIMENTAL

### Strains, plasmids and media

The yeast strains and plasmids used are shown in Tables 1 and 2 respectively. The cDNA library was constructed by Invitrogen, using mRNA isolated from mouse brain. Double-stranded cDNA was cloned in a Gateway Topo cloning vector and the library was swapped into the pVV214 yeast expression vector [20]. αSyn is expressed from the *GAL1* promoter, either on the multi-copy plasmid pESC-HIS (Agilent Technologies) or integrated in two copies in the genome, via integrative vectors pRS403 and pRS405 (Stratagene).

**Table 1:** Plasmids used in the present study *H.s.*, *Homo sapiens*; *M.m.*, *Mus musculus*; *S.c.*, *Saccharomyces cerevisiae*.

Plasmid name	Vector	Type	Selection marker	Coding sequence*
pESC/αSyn	pESC-His	Multi-copy	*HIS3*	*H.s.* αSyn cDNA (WT)
pESC/αSynA53T	pESC-His	Multicopy	*HIS3*	*H.s.* αSyn cDNA (A53T)
pESC/αSynA30P	pESC-His	Multicopy	*HIS3*	*H.s.* αSyn cDNA (A30P)
pESC/αSyn-GFP	pESC-His	Multi-copy	*HIS3*	*H.s.* αSyn cDNA (WT)–yeGFP C-terminal fusion
pRS403/αSyn	pRS403	Integrative	*HIS3*	*H.s.* αSyn cDNA (WT)
pRS403/αSyn-GFP	pRS403	Integrative	*HIS3*	*H.s.* αSyn cDNA (WT)–yeGFP C-terminal fusion
pRS405/αSyn	pRS405	Integrative	*LEU2*	*H.s.* αSyn cDNA (WT)
pRS405/αSyn-GFP	pRS405	Integrative	*LEU2*	*H.s.* αSyn cDNA (WT)- yeGFP C-terminal fusion
Empty vector	pVV214	Multi-copy	*URA3*	
pPSMA2	pVV214	Multi-copy	*URA3*	*M.m* PSMA2 cDNA †
pRPS3A	pVV214	Multi-copy	*URA3*	*M.m.* RPS3A cDNA †
pSpir1CT	pVV214	Multi-copy	*URA3*	*M.m.* Spire homologue 1 cDNA amino acids 266–258*
pRPS1A	pVV214	Multi-copy	*URA3*	*S.c.* ORF *YLR441C*†
pRPS1B	pVV214	Multi-copy	*URA3*	*S.c.* ORF *YML063W*†
pRPS3AΔ2-50	pVV214	Multi-copy	*URA3*	*M.m.* RPS3A cDNA M1, amino acids 51–264
pRPS3AΔ164-264	pVV214	Multi-copy	*URA3*	*M.m.* RPS3A cDNA amino acids 1–163
pRPS3AΔ114-264	pVV214	Multi-copy	*URA3*	*M.m.* RPS3A cDNA amino acids 1–113
pRPS3AΔ51-264	pVV214	Multi-copy	*URA3*	*M.m.* RPS3A cDNA amino acids 1–50

*Isolated from a mouse brain cDNA library in the screening (see the text for details).

†From the FLEXGene yeast protein-coding clone collection [41], Gateway® cloned.

**Table 2:** Yeast strains used in the present study

Strain name	Genotype	Reference
BY4742	*MAT*α *his3*Δ*1 leu2*Δ*0 lys2*Δ*0 ura3*Δ*0*	[42]
*cog6*Δ	BY4742 *cog6*Δ*::KANMX4*	[43]
*vps52*Δ	BY4742 *vps52*Δ*::KANMX4*	[43]
*dpp1*Δ	BY4742 *dpp1*Δ*::KANMX4*	[43]
*opi3*Δ	BY4742 *opi3*Δ*::KANMX4*	[43]
*sod2*Δ	BY4742 *sod2*Δ*::KANMX4*	[43]
*vps24*Δ	BY4742 *vps24*Δ*::KANMX4*	[43]
BY2E	BY4742 *his3*Δ*1*::pRS403 *leu2*Δ*0*::pRS405	The present study
BY2αSyn	BY4742 *his3*Δ*1*::pRS403/αSyn *leu2*Δ*0*::pRS405/αSyn	The present study
*cog6*Δ2E	BY4742 *cog6*Δ*::KANMX4 his3*Δ*1*::pRS403 *leu2*Δ*0*::pRS405	The present study
*cog6*Δ2αSyn	BY4742 *cog6*Δ*::KANMX4 his3*Δ*1*::pRS403/αSyn *leu2*Δ*0*::pRS405/αSyn	The present study
*cog6*Δ2αSyn-GFP	BY4742 *cog6*Δ*::KANMX4 his3*Δ*1*::pRS403/αSyn-GFP *leu2*Δ*0*::pRS405/αSyn-GFP	The present study
*rps1a*Δ	BY4742 *rps1a*Δ*::KANMX4*	[43]
*rps1b*Δ	*MAT*a *his3*Δ*1 leu2*Δ*0 met15*Δ*0 ura3*Δ*0 rps1b*Δ*::KANMX4*	[43]
*rps1a*Δ/*rps1b*Δ	BY*rps1a*Δα/BY*rps1b*Δa	The present study

Cells were pre-grown in non-inducing raffinose (2% w/v) selective medium (SC−His, SC−Ura, SC−Leu−His, SC−Ura−Leu−His, where SC is synthetic complete lacking histidine, uracil, leucine and histidine, and uracil, leucine and histidine respectively; BIO101) and then shifted to galactose (2% w/v) selective medium for induction of αSyn expression. For microscopy, cells were grown in raffinose (2% w/v) selective medium, after which they were transferred to galactose (2% w/v) selective medium for 15 h. For sporulation, sodium acetate (0.5%) agar plates were used. For loss of pVV214-derived plasmids, agar plates (SC, 2% glucose) with 0.1% 5-FOA (5-fluoro-orotic acid) were used. To test for the presence of the KanMX gene-disruption cassette, cells were streaked on to YPD (yeast extract/peptone/dextrose: 1% yeast extract, 2% peptone, 2% glucose) agar plates containing 300 mg/l geneticin (G418, Invitrogen).

### Growth assays

Yeast cells were grown in raffinose minimal medium to mid-exponential phase at 30°C. A dilution series (*D*_600_ = 1, 0.1, 0.01 and 0.001) was made with sugar-free minimal medium. Then, 5 μl of each dilution was spotted on to agarose plates with galactose or glucose minimal medium, which were incubated at 30°C. The assays were evaluated at 1, 2 and 3 days, but the results were qualitatively similar and quantitatively clearer at 3 days. Hence the latter are shown in the Figures. For all direct comparisons between strains or plasmids, the growth assays were done on one and the same agarose plate. For growth assays in liquid media, cells were also cultured in raffinose minimal medium to mid-exponential phase at 30°C and growth curves were determined in liquid minimal galactose medium, inoculated at *D*_600_ = 0.05 and incubated at 30°C. *D*_600_ was measured every 1 h for 3 days in a Bioscreen C System. αSyn toxicity was enhanced in WT cells by the addition of 9% (v/v) DMSO.

### Screening of the cDNA library

The *cog6*Δ2αSyn strain was transformed with the cDNA library after which the cells were plated out on galactose. From the transformants obtained, those that could not grow on plates containing galactose (for induction of αSyn expression) and 5-FOA (for loss of the cDNA plasmid) were selected for an αSyn toxicity test. From the colonies that showed good growth, the cDNA plasmids were isolated and retransformed in the *cog6*Δ2αSyn strain. With these transformants the αSyn toxicity test was repeated to confirm the suppression of αSyn toxicity by the cDNA plasmids isolated.

### Western blot analysis

Cells were grown at 30°C in selective raffinose medium until they reached exponential phase. They were then transferred for 15 h to galactose medium to induce αSyn expression. Protein extracts were obtained as described in [21]. Mouse brain extract in SDS/PAGE loading buffer was obtained from Sigma–Aldrich. SDS/PAGE was performed using Invitrogen NuPAGE Novex 4–12% Bis-Tris Gels. αSyn and αSyn–GFP protein levels were determined by Western blot analysis using a rabbit anti-αSyn antibody (Cell Signaling Technology), diluted 1:500, and compared with Pgk1 (phosphoglycerate kinase 1) levels determined by Western blotting using a mouse anti-Pgk1 antibody (Molecular Probes), diluted 1:500, or to Gapdh (glyceraldehyde-3-phosphate dehydrogenase) using a mouse anti-Gapdh antibody (Millipore), diluted 1:1000. Secondary antibodies used were HRP (horseradish peroxidase)-conjugated donkey anti-(rabbit IgG) ECL antibody and HRP-conjugated sheep anti-(mouse IgG) ECL antibody (GE Healthcare), both diluted 1:5000. RPS3A protein levels were determined by Western blot analysis using a custom-made affinity-purified chicken IgY polyclonal antibody (Genway), diluted 1:1000. As the secondary antibody HRP-conjugated goat anti-(chicken IgY) Fc fragment (Genway) was used, diluted 1:5000.

### Complementation analysis

The heterozygous diploid strain *rps1a*Δ*/RPS1A rps1b*Δ*/RPS1B* (obtained by mating of *rps1a*Δα with *rps1b*Δa) was transformed with a plasmid, after which transformants were sporulated (as described previously [22]) and growth of the segregants was assessed on SD (synthetic dextrose) lacking uracil (SD−Ura), SD plates containing 5-FOA, a medium where loss of the *URA3*-encoding plasmids is induced, and on YPD plates with geneticin, where only the segregants that contain at least one KanMX-disrupted gene can grow. The genotypes of the segregants were confirmed by PCR on the genomic DNA.

### Fluorescence microscopy

Laser-scanning microscopy was performed with an LSM510 system (Carl Zeiss), using Koehler illumination and a high-NA (numerical aperture) objective (C-Apochromat 40×/NA1.2/W). Yeast cell cultures were placed between two 100-μm-thick cover slips. The 488-nm line of an argon-ion laser (acousto-optical tunable filter 15%) was used to excite yeGFP (yeast-enhanced GFP). Fluorescence of yeGFP was captured through a BP505-530 filter.

## RESULTS

### Establishment of a yeast screening system for isolation of suppressors of αSyn toxicity

**Figure 1:**
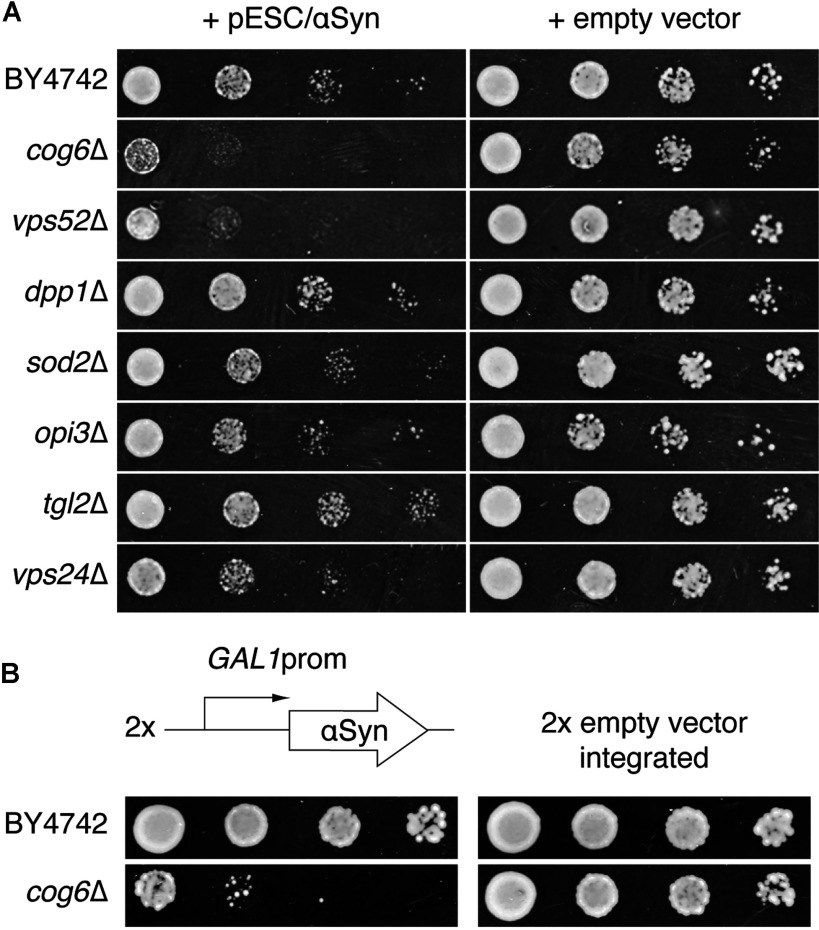
The *cog6*Δ strain is highly sensitive to αSyn expression (**A**) Yeast strains BY4742, *cog6*Δ, *vps52*Δ, *dpp1*Δ, *sod2*Δ, *opi3*Δ, *tgl2*Δ and *vps24*Δ were transformed with the pESC/αSyn plasmid for galactose-induced expression of αSyn or empty vector pESC−His and a growth assay on SC−His plates containing galactose was performed as described in the Experimental section. (**B**) Two copies of human WT αSyn, under the control of the *GAL1* promoter, or empty vectors pRS403 and pRS405 were integrated into the genome of the BY4742 and *cog6*Δ strains. A growth assay on SC−Leu−His plates containing galactose was performed as described in the Experimental section.

We have tested yeast deletion strains reported to be sensitive to αSyn expression [12,23] for those that showed the highest sensitivity, in order to minimize possible artefacts due to high overexpression of αSyn in the yeast model system. For that purpose, we transformed the strains with a multi-copy vector containing human WT αSyn (NCBI accession number AAI08276) under the control of a galactose-inducible promoter and tested the transformants for residual growth on selective galactose medium. Under the conditions used in the present study αSyn expression caused, at most, a slight growth inhibition of the *dpp1*Δ, *opi3*Δ, *sod2*Δ, *tgl2*Δ and *vps24*Δ strains and isogenic WT strain BY4742. A strong growth defect was observed in the *vps52*Δ strain, but the *cog6*Δ strain was most sensitive to galactose-induced expression of αSyn (Figure 1A). To obtain a stable cell line for screening purposes we have integrated two galactose-inducible copies of the αSyn ORF into the genome of the *cog6*Δ strain. The resulting transformants (*cog6*Δ2αSyn) showed a strong growth defect on galactose (Figure 1B). On glucose all strains grew well and also cells transformed with empty vectors showed the same growth as untransformed cells (results not shown). Hence we used the *cog6*Δ2αSyn strain in the subsequent screening for mammalian suppressor clones. In contrast with the observations of Outeiro and Lindquist [24], we did not observe an αSyn-induced growth defect in our WT strain with two galactose-inducible copies of αSyn integrated in the genome.

### Screening for suppressors of αSyn toxicity with a mouse brain cDNA library

**Figure 2:**
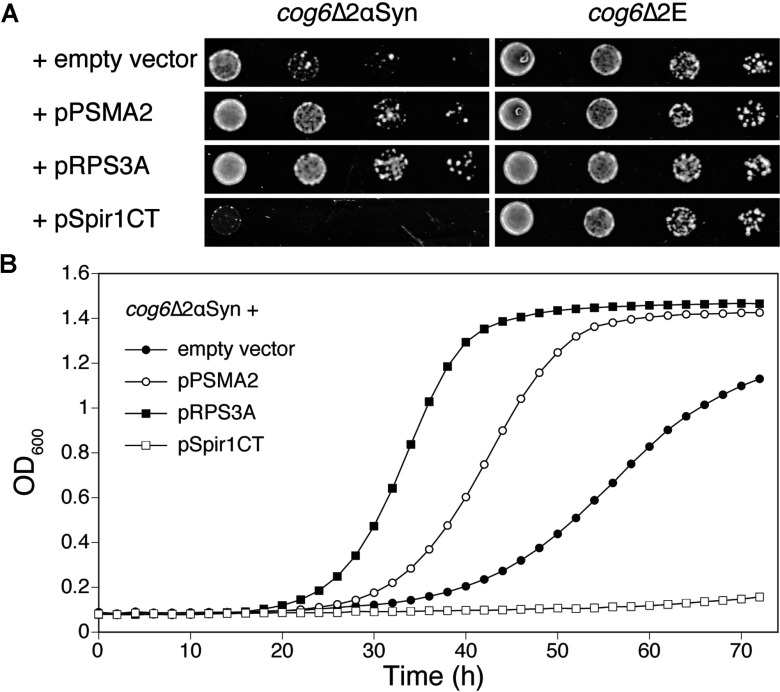
Two mammalian suppressors and one enhancer of αSyn toxicity have been identified (**A**) Strain *cog6*Δ2αSyn and the control strain not expressing αSyn were transformed with empty vector pVV214 or with the mouse cDNA plasmids isolated in the screening, as indicated on the Figure. A growth assay was performed on SC−Ura−Leu−His galactose plates, as described in the Experimental section. (**B**) Growth curves were determined in SC−Ura−Leu−His galactose (2% w/v) medium, using a Bioscreen C device as described in the Experimental section. Strains: *cog6*Δ2αSyn+empty vector (●), pPSMA2 (○), pRPS3A (■), pSpir1CT (□).

Total mRNA was isolated from mouse brain and used for construction of a cDNA library, custom-made by Invitrogen. The cDNA library was subcloned into the yeast expression vector pVV214 [20] and the resulting library was transformed into the *cog6*Δ2αSyn strain. We obtained approximately 36000 independent transformants of which approximately 500 transformants were able to grow on the galactose-containing plates. Only the colonies that did not grow on galactose after loss of the cDNA (*URA3*) plasmid on medium with 5-FOA, a substance converted into the toxic fluorouracil by orotine 5′-monophosphate decarboxylase, the *URA3* gene product [25], were retained for plasmid isolation. After retransformation of the isolated plasmids in the *cog6*Δ2αSyn strain, we found two clones that strongly reduced the growth defect of *cog6*Δ2αSyn cells on αSyn-inducing medium: they encoded the mouse ribosomal protein S3A (pRPS3A, NCBI accession number NP_058655) and the proteasome subunit α type 2 (pPSMA2, NCBI accession number NP_032970). One of the isolated cDNA plasmids surprisingly enhanced cell death upon retransformation in the *cog6*Δ2αSyn strain (Figure 2A). This cDNA plasmid had no growth-inhibitory effect in the *cog6*Δ strain without αSyn, indicating that the cDNA product apparently enhances αSyn toxicity. This plasmid encodes a fragment of the actin organizer protein Spire homologue 1 (NCBI accession number NP_919336) containing the 263 C-terminal amino acids (termed pSpir1CT). The three plasmids had a corresponding effect on the growth rate of *cog6*Δ2αSyn cells in liquid galactose medium (Figure 2B). As a Bioscreen C device was used for the attenuance readings, the measurements are saturated at a *D*_600_ of approximately 1.4. Hence this does not indicate that the cells have reached stationary phase.

### RPS3A suppresses αSyn toxicity in other backgrounds than the *cog6*Δ strain, but does not influence αSyn protein levels

**Figure 3:**
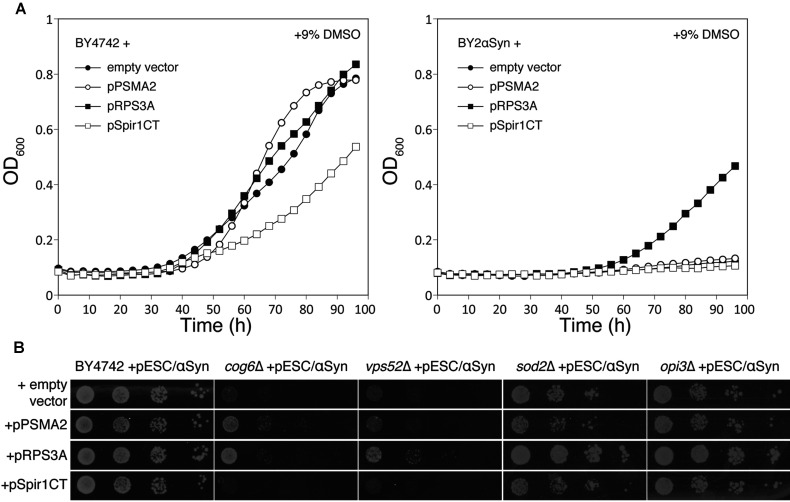
PS3A counteracts the αSyn-induced growth defect irrespective of the genetic background (**A**) RPS3A counteracts DMSO-enhanced toxicity of αSyn and has no effect on DMSO toxicity in a strain not expressing αSyn, PSMA2 does not influence the DMSO-enhanced αSyn toxicity and Spir1CT enhances the DMSO-induced growth defect independent of αSyn expression. Left-hand panel: BY4742 without αSyn; right-hand panel: BY2αSyn. Plasmids: empty vector (●), pPSMA2 (○), pRPS3A (■), pSpir1CT (□). Growth curves in SC−Ura galactose medium with 9% DMSO were obtained as described in the Experimental section. (**B**) RPS3A suppresses αSyn toxicity in αSyn-sensitive strains *cog6*Δ, *vps52*Δ, *sod2*Δ and *opi3*Δ. PSMA2 only affects the growth of αSyn-expressing *cog6*Δ cells. Spir1CT does not enhance the weak αSyn-sensitivity of strains BY4742, *sod2*Δ or *opi3*Δ. Transformants, as indicated on the Figure, were spotted on to SC−Ura−His galactose plates as described in the Experimental section.

A drawback of doing the screening in a sensitive mutant strain is that suppressors isolated could be acting on the sensitivity of that strain, rather than reducing the toxicity of αSyn. To address this issue, we investigated whether the observed effects were also present in other backgrounds. Because no clear effects of αSyn or the other brain proteins could be observed in WT BY4742 cells grown in normal inducing medium, we performed growth experiments in the presence of DMSO. Addition of DMSO was reported to enhance the amount of αSyn-induced inclusions, leading to growth reduction [26]. Addition of 9% (v/v) DMSO caused a much stronger growth inhibition of cells expressing αSyn than in cells without αSyn, but only RPS3A counteracted the DMSO-induced growth inhibition of the BY2αSyn strain, and it had no effect in the absence of αSyn. PSMA2 did not affect the growth of either strain with or without αSyn or DMSO, whereas Spir1CT enhanced the DMSO-induced growth defect, independent of αSyn expression (Figure 3A). RPS3A also improved the growth of BY2αSyn in the presence of 9 mM ZnSO_4_, which was demonstrated to increase αSyn toxicity [27] (results not shown). When we tested the suppressors and enhancer in other αSyn-sensitive strains (Figure 3B), we observed that PSMA2 only suppresses the αSyn-sensitivity of the *cog6*Δ strain, whereas RPS3A also counteracted the αSyn-induced growth defect in the strains *vps52*Δ, *sod2*Δ and *opi3*Δ. For Spir1CT, we did not observe an increased growth defect in the strains with weak αSyn-sensitivity: BY4742, *sod2*Δ and *opi3*Δ. We conclude that only RPS3A is directly affecting αSyn toxicity, whereas PSMA2 is a suppressor of the αSyn-sensitivity in the *cog6*Δ background and the C-terminus of Spire homologue 1 enhances lethality in cells that are challenged with various stresses, but not specifically αSyn-induced lethality.

**Figure 4:**
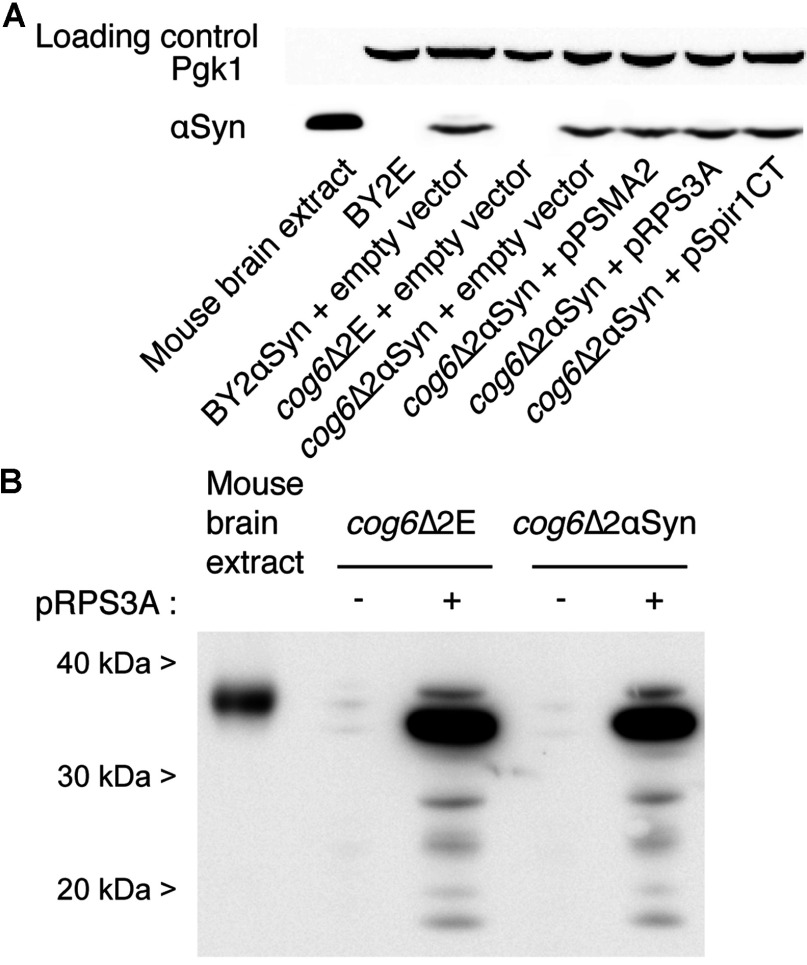
Western blot experiments of mammalian proteins expressed in yeast (**A**) Deletion of *COG6* and co-expression of brain proteins do not significantly change expression levels of αSyn. Western blot analysis 15 h after induction on galactose (2% w/v). Primary antibodies: (top) anti-yeast Pgk1 (produced in mouse) as a loading control, (bottom) anti-human αSyn (produced in rabbit). (**B**) αSyn expression does not change the appearance of RPS3A in SDS/PAGE. Western blot analysis 15 h after induction on galactose (2% w/v). Primary antibody: anti-mouse RPS3A (produced in chicken). The molecular mass in kDa is indicated on the left-hand side.

None of the isolated mouse cDNAs, nor the deletion of *COG6*, significantly influenced αSyn protein levels, as judged by Western blotting experiments (Figure 4A). Mouse αSyn from a commercially available mouse brain extract (Sigma) migrated slightly higher than the yeast-expressed human αSyn, possibly due to the seven-amino-acid differences between the two proteins. A custom-made antibody against RPS3A (from Cell Signaling Technology) readily detected the yeast-expressed RPS3A (Figure 4B). Both in the yeast extracts and in the mouse brain extract from Sigma, mouse RPS3A migrated approximately 6 kDa higher than its predicted molecular mass of 29.89 kDa. Several lower-molecular-mass bands were present in the yeast extracts from yeast cells with pRPS3A, and the strongest band was the second largest form, approximately 2 kDa smaller than the form present in the mouse brain extract from Sigma. Very weak bands of similar sizes can be seen in the extracts from cells carrying the empty vector instead of pRPS3A, probably due to cross-reactivity of the antibody with the yeast homologues of RPS3A. There are no indications that the presence of αSyn influences the protein levels or the post-translational processing of RPS3A. Despite several attempts using both the specific antibodies and an anti-c-Myc antibody against a Myc-tagged version of αSyn, we were unable to reproducibly demonstrate co-immunoprecipitation of RPS3A with αSyn, or vice versa.

### Mouse RPS3A suppresses αSyn A53T toxicity in yeast

**Figure 5:**
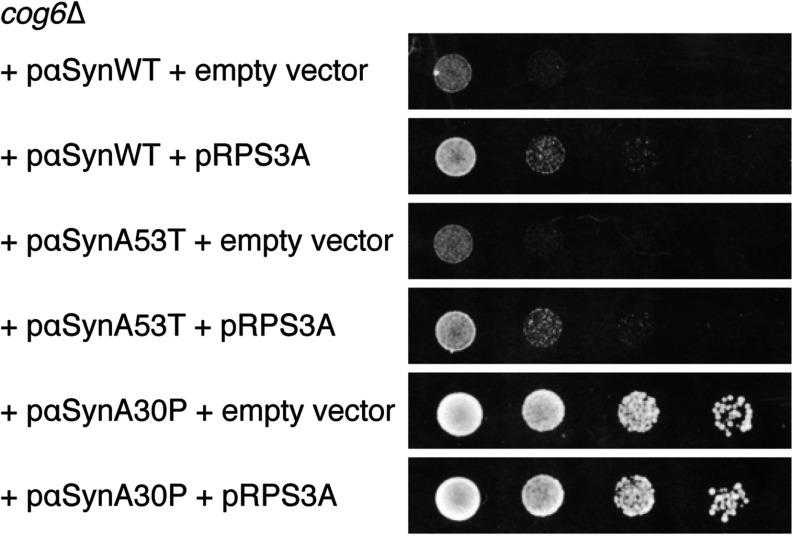
RPS3A counteracts αSyn A53T toxicity and αSyn A30P is not toxic in *cog6*Δ yeast cells Strain *cog6*Δ was transformed with the plasmids indicated. A spot dilution growth assay was performed on SC−Ura−His galactose plates, as described in the Experimental section.

It was shown previously that, when expressed in yeast, αSyn A53T induces a growth defect similar to αSyn WT. In contrast with WT and A53T, αSyn A30P does not localize to the plasma membrane, but remains dispersed throughout the cytoplasm and does not induce a growth defect in yeast [24]. When expressed in yeast strain *cog6*Δ, we observed that αSyn A53T causes a strong growth defect that is suppressed by co-expression of RPS3A. The expression of αSyn A30P does not result in a growth defect in the *cog6*Δ background (Figure 5).

### Mouse RPS3A complements the deletion of its yeast homologues RPS1A and RPS1B, but the yeast homologues do not suppress the *cog6*Δ2αSyn growth defect

**Figure 6:**
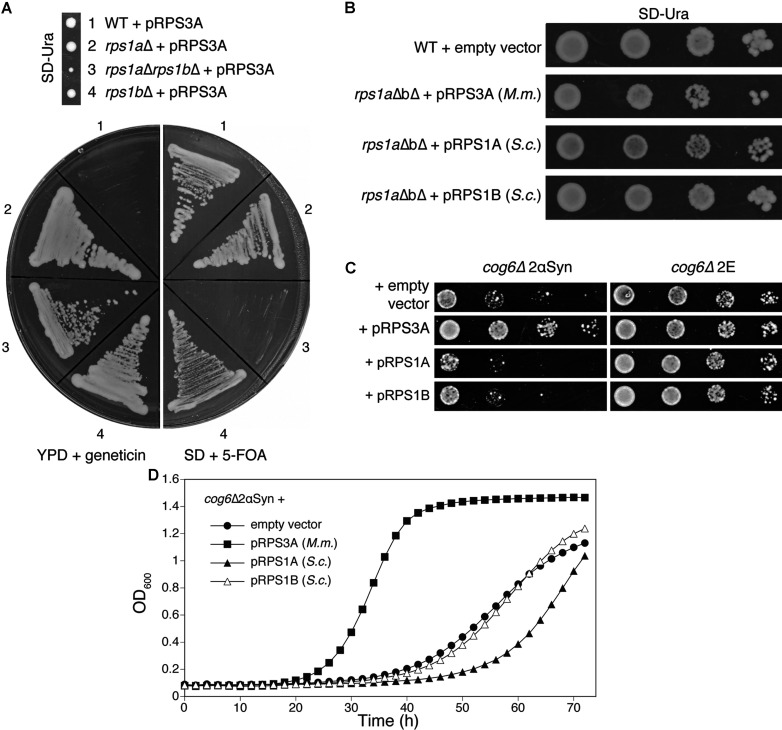
Plasmids pRPS3A (mouse) and pRPS1A and pRPS1B (yeast) complement synthetic lethality of *rps1a*Δ *rps1b*Δ double deletion, but only pRPS3A suppresses the αSyn-induced growth defect (**A**) Heterozygous diploid strain *rps1a*Δ*/RPS1A rps1b*Δ*/RPS1B* was obtained by crossing *rps1a*Δ with *rps1b*Δ. After transformation of this strain with pRPS3A, sporulation and tetrad dissection, the segregants were streaked on to YPD plates with geneticin and SD plates with 5-FOA. (**B**) *rps1a*Δ *rps1b*Δ cells carrying pRPS1A or pRPS1B were obtained by tetrad dissection of transformed heterozygous diploids. A growth assay on SD−Ura plates was performed with the indicated strains as described in the Experimental section. *M.m.*, *Mus musculus*; *S.c.*, *Saccharomyces cerevisiae*. (**C**) Strain *cog6*Δ2αSyn and the control strain not expressing αSyn were transformed with the indicated plasmids. A growth assay was performed on SC−Ura−Leu−His galactose plates, as described in the Experimental section. (**D**) Growth curves were determined in SC−Ura−Leu−His galactose (2% w/v) medium, using a Bioscreen C device as described in the Experimental section. Strains: *cog6*Δ2αSyn + empty vector (●), pRPS3A (■), pRPS1A (▲), pRPS1B (Δ).

Mammalian RPS3A has two homologous proteins in yeast, Rps1A and Rps1B. We found that double deletion of the corresponding genes is lethal and that pRPS3A can overcome the lethality of the *rps1a*Δ *rps1b*Δ strain (Figure 6A). This indicates that mouse RPS3A probably contributes to the ribosome activity of the yeast. We have cloned the yeast homologues into the pVV214 vector (pRPS1A and pRPS1B, NCBI accession numbers NP_013546 and NP_013648), and evaluated the capacity of the resulting plasmids to complement the lethality of the *rps1a*Δ *rps1b*Δ deletion strain. Complementation was observed and *rps1a*Δ *rps1b*Δ+pRPS1A or pRPS1B even grew slightly better than *rps1a*Δ *rps1b*Δ +pRPS3A (Figure 6B), indicating that all three plasmids allow the cells to produce functional ribosomal subunits. When we transformed plasmids pRPS1A and pRPS1B into the *cog6*Δ2αSyn strain and assessed growth on galactose, they did not suppress αSyn-induced lethality, neither on agar plates (Figure 6C) nor in liquid medium (Figure 6D). pRPS1A even seemed to slightly enhance the αSyn-induced growth defect. Again, the plateau reached in the growth curves is due to saturation of the Bioscreen C reading at this *D*_600_ value, which should not be interpreted as the cells entering stationary phase. We can conclude that the suppression of αSyn toxicity by RPS3A is a specific function of the mammalian protein, which is absent from its yeast counterparts.

### Identification of the domain of RPS3A necessary for suppression of αSyn toxicity in yeast

**Figure 7:**
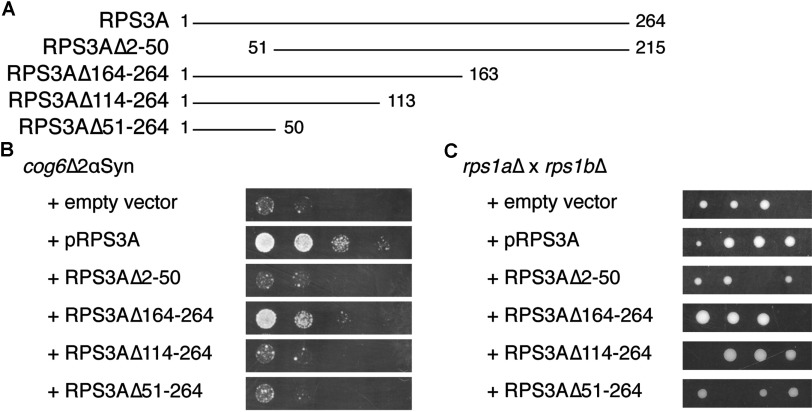
The 50 N-terminal amino acids of RPS3A are essential for its suppression effect on αSyn toxicity; truncation of 101 C-terminal amino acids results in residual activity (**A**) Schematic representation of the truncated alleles of RPS3A that were cloned into vector pVV214 for the present study. (**B**) Truncation of the 50 N-terminal amino acids of RPS3A abolishes its capacity to counteract αSyn toxicity in strain *cog6*Δ2αSyn, whereas deletion of the 101 C-terminal amino acids results in residual suppression activity; deletion of larger C-terminal domains (151 or 214 amino acids) results in the absence of αSyn toxicity suppression. Strain *cog6*Δ2αSyn was transformed with the indicated plasmids and a growth assay was performed on SC−Ura−Leu−His galactose plates as described in the Experimental section. (**C**) None of the truncated versions of RPS3A can complement synthetic lethality of the *rps1a*Δ *rps1b*Δ strain. Heterozygous diploid strain *rps1a*Δ*/RPS1A rps1b*Δ*/RPS1B* was transformed with the indicated plasmids, transformants were sporulated and tetrad dissection was performed on SD−Ura plates.

In the study by Lim et al. [18] it was shown that 50 N-terminal amino acids of RPS3A are essential to interact with and to increase the solubility of the HBx (hepatitis B protein X) in hepatocellular carcinoma cells, whereas from the C-terminus up to 101 amino acids could be deleted without affecting this chaperone function. We have created a yeast expression clone in which the coding sequence for the 50 N-terminal amino acids except the start codon was removed (pRPS3AΔ2-50) as well as clones for expression of C-terminal deletions of 101 amino acids (pRPS3AΔ164-264), 151 amino acids (pRPS3AΔ114-264) and 214 amino acids (pRPS3AΔ51-264), as represented in Figure 7(A). By expressing these plasmids in our yeast strain, we have evaluated which domain is essential and which is sufficient for suppression of αSyn toxicity in yeast. From the growth assay (Figure 7B), we can conclude that the 50 N-terminal amino acids are indeed essential for this function of RPS3A, as pRPS3AΔ2-50 has lost the ability to suppress αSyn toxicity. However, they are not sufficient because pRPS3AΔ51-264 also does not result in restored growth of *cog6*Δ2αSyn on galactose. Because plasmid pRPS3AΔ164-264 still improves growth on galactose (albeit to a lesser extent than full-length RPS3A) but pRPS3AΔ114-264 does not, we conclude that amino acids 51–164 are also essential for the suppression of αSyn toxicity.

To evaluate whether the truncations affect the ribosomal function of RPS3A, we have transformed the heterozygous diploid strain *RPS1A/rps1a*Δ *RPS1B/rps1b*Δ with the different plasmids and sporulated the transformants to evaluate which of the plasmids could complement the synthetic lethality of the *rps1a*Δ *rps1b*Δ strain. Only when the plasmid expressing full-length RPS3A was transformed, were four growing spores obtained (Figure 7C), indicating that none of the truncation mutants had retained the ribosomal function of RPS3A.

### Mouse RPS3A and RPS3AΔ164-264 reduce αSyn–GFP inclusion formation in the *cog6*Δ2αSyn-GFP strain

**Figure 8:**
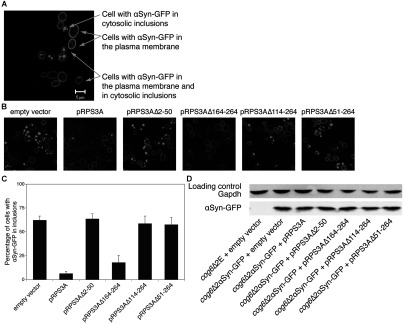
RPS3A and RPS3AΔ164-264 counteract αSyn–GFP inclusion formation in strain *cog6*Δ2αSyn-GFP (**A**) Three αSyn–GFP localization patterns are observed, i.e. cells with only plasma membrane localization, cells with only cytosolic inclusions of αSyn–GFP and cells with both localizations. (**B**) Sample images of GFP localization in *cog6*Δ2αSyn-GFP cells transformed with the indicated plasmids, 15 h after induction on galactose (2% w/v). (**C**) Approximately 200 cells of each strain were counted and divided according to the presence of inclusions. The effect of the plasmids indicated on the localization pattern is shown in the histograms. Values are means±S.E.M. obtained by repeating the experiment with three independent transformants. (**D**) Co-expression of RPS3A or one of its truncation forms does not influence αSyn–GFP protein levels. Western blot analysis, 15 h after induction with galactose (2% w/v). Primary antibodies: (top) anti-yeast Gapdh (produced in mouse) as a loading control; (bottom) anti-human αSyn (produced in rabbit).

A previous study showed that αSyn–GFP constructs in yeast are not subject to proteolysis, as opposed to related fusions in mammalian cells [12], and that the fusion with GFP does not influence the localization of αSyn. Hence we integrated two copies of a construct encoding galactose-inducible αSyn–GFP into the genome of the *cog6*Δ strain, resulting in the strain *cog6*Δ2αSyn-GFP. The C-terminal fusion of GFP to αSyn did not influence its toxicity in the *cog6*Δ background and RPS3A and RPS3AΔ164-264 also suppressed αSyn–GFP toxicity in the *cog6*Δ2αSyn-GFP strain, whereas the other truncations did not (results not shown). We studied the intracellular localization of αSyn as a function of time after induction of αSyn–GFP expression with galactose and observed three patterns of intracellular localization; at early time points αSyn–GFP was located nearly exclusively at the plasma membrane, later on (starting 6 h after the induction of αSyn expression) part of the cells showed both localization at the plasma membrane and in cytosolic inclusions and finally (starting 12 h after the induction of αSyn expression) some cells showed αSyn–GFP fluorescence exclusively in cytosolic inclusions (Figure 8A). Co-expression of RPS3A and RPS3AΔ164-264 clearly delays the shift of αSyn–GFP from the plasma membrane to the cytosolic inclusions, whereas cells transformed with the other truncation mutants behave much like empty-vector-transformed cells (Figure 8B). These effects were quantified by counting the cells with αSyn–GFP localized in inclusions, 15 h after induction of αSyn–GFP expression with galactose (Figure 8C). A Western blot was performed with cell extracts from these strains and showed that co-expression of RPS3A or its truncation forms does not influence αSyn–GFP expression levels (Figure 8D). Although it has been shown previously that inclusion formation is not a prerequisite for αSyn-induced toxicity in yeast [23,28,29], we observe here that in the *cog6*Δ2αSyn-GFP strain, toxicity is strongly correlated with the extent of cytosolic inclusion formation and that RPS3A counteracts this. As for the suppression of the αSyn-induced growth defect, deletion of the 50 N-terminal amino acids abolishes the effect, whereas RPS3A with 101 C-terminal amino acids deleted retains much of the activity against inclusion formation.

## DISCUSSION

### The *cog6*Δ2αSyn screening system was used to identify mammalian brain proteins that suppress αSyn toxicity in yeast

Efficient screening of the mouse brain cDNA library in yeast depended on a strong αSyn-induced toxicity phenotype. To avoid excessive overexpression of αSyn, we opted for using a deletion mutant with increased sensitivity to αSyn overexpression. In our hands, the strains *dpp1*Δ, *opi3*Δ, *sod2*Δ, *tgl2*Δ and *vps24*Δ were only slightly or not at all sensitive to αSyn expressed from the *GAL1* promoter on a multi-copy plasmid, in contrast with what was observed in a previously reported screening of the yeast deletion collection [12]. The sensitivity of the strains *cog6*Δ and *vps52*Δ to αSyn expression was much higher, and integration of two galactose-inducible copies of WT human αSyn in the strain *cog6*Δ (*cog6*Δ2αSyn) resulted in a sufficiently strong growth defect allowing efficient screening of the brain cDNA library for suppressors of αSyn-induced toxicity. The advantage in using such a sensitive strain for the cDNA screening is that a toxicity phenotype can be obtained with much lower αSyn expression levels. In this way, we can avoid isolating suppressors that may just counteract toxicity caused by a high overload of foreign protein or due to irrelevant toxicity caused by very high αSyn levels. Cog6 is a non-essential component of the COG complex, a cytosolic tethering complex that functions in protein trafficking to mediate fusion of transport vesicles to Golgi compartments [30]. Cog6 is proposed to mediate the interaction of the COG complex with t-SNARE (target SNARE) proteins [31,32]. Apparently, the Cog6 protein plays an essential role in the resistance of WT *S. cerevisiae* cells to αSyn toxicity. The fact that we did not observe αSyn-induced lethality in a WT yeast strain with two galactose-inducible copies of αSyn integrated, whereas Outeiro and Lindquist [24] describe a strong growth defect, can be explained by differences in the αSyn expression levels due to different chromosomal integration sites, or by differences in the genetic background of the strains (BY4742 instead of W303-1A).

Transforming the *cog6*Δ2αSyn strain with the mouse-brain-specific cDNA library, elimination of false positives using 5-FOA and confirmation by retransformation and growth assays resulted in the identification of two suppressors of the αSyn-induced growth defect: the mouse PSMA2 and RPS3A. One of the isolated cDNA plasmids surprisingly enhanced the growth defect upon retransformation in the *cog6*Δ2αSyn strain, but it had no growth-inhibitory effect in the *cog6*Δ strain without αSyn, indicating that the cDNA product apparently enhances αSyn toxicity. This cDNA encodes a fragment of the Spire homologue 1 protein, containing the 263 C-terminal amino acids (Spir1CT).

A drawback of the use of the *cog6*Δ2αSyn strain for the screening is that some of the hits may not represent suppressors of the αSyn-induced toxicity, but rather those of the *cog6*Δ-induced sensitivity. This can be easily controlled by assessing the effects of the isolated suppressors on αSyn-induced growth defects in yeast strains with a different background. As PSMA2 could only suppress the growth defect of *cog6*Δ cells, but not of *vps52*Δ, *sod2*Δ, *opi3*Δ or BY2αSyn cells challenged with DMSO, we had to conclude that PSMA2 is a suppressor of the *cog6*Δ-induced sensitivity. Apart from the fact that PSMA2 does not alter the αSyn protein levels in strain *cog6*Δ2αSyn, we have no lead on the mechanism by which PSMA2 counteracts the sensitivity induced by the deletion of *COG6.* Similarly, the C-terminus of Spire homologue 1 could not enhance the mild αSyn toxicity in BY4742, *sod2*Δ and *opi3*Δ, but did increase the growth defect of the BY4742 strain challenged with DMSO in the absence of αSyn. Plasmid pSpir1CT is thus an enhancer of the lethality in yeast strains challenged with various stresses, but not specific for αSyn. Kerkhoff et al. [33] describe how overexpression of an N-terminally truncated form of mouse Spire homologue 1, a protein only 37 amino acids shorter than the protein encoded by the cDNA we isolated, in a murine fibroblast 3T3 cell line strongly inhibited the transport of the VSV-G (vesicular stomatitis virus G) protein to the plasma membrane and how a transport step in the exocytosis pathway was blocked. Overexpression of the full-length Spire homologue 1 had no effect [33]. Similarly, a clone encoding the full-length cDNA of Spire homologue 1 did not enhance αSyn-induced growth inhibition in the *cog6*Δ2αSyn strain (results not shown).

### Mammalian RPS3A suppresses αSyn toxicity and counteracts αSyn–GFP inclusion formation in yeast cells

The ribosome has no well-studied link with αSyn misfolding or aggregation. However, there are some studies that show a possible link of RPS3A with neurodegeneration. One group found that *Rps3a* gene expression is up-regulated in mice treated with MPTP (1-methyl-4-phenyl-1,2,3,6-tetrahydropyridine) [34]. Furthermore, RPS3A protein levels are significantly higher in the striatum of MPTP- and methamphetamine-treated mice than in control mice [35]. One genetic linkage study claimed that a mutation in *RPS3A*, SNP *rs498055*, is linked to late-onset Alzheimer's disease [36]; however, that conclusion was later contradicted by three other studies [37–39].

In the present study we show that mouse RPS3A (which differs only in two amino acids from human RPS3A) can counteract toxicity caused by human WT αSyn, in several αSyn-sensitive yeast deletion mutants, as well as in WT cells treated with DMSO or ZnSO_4_, a strong indication that RPS3A directly affects αSyn, rather than reducing the sensitivity of the yeast cells. RPS3A was also shown to suppress the yeast growth defect caused by αSyn A53T. The suppression activity is not related to the synthesis or breakdown of αSyn, as indicated by Western blot analysis of the αSyn protein content. We observed that the most prominent band for RPS3A in Western blot analysis of yeast extracts is approximately 2 kDa smaller than the band detected in mouse brain extracts, which could indicate some post-translational processing in yeast which does not occur in mouse brain cells, or vice versa.

The fact that we failed to reproducibly demonstrate co-immunoprecipitation between RPS3A and αSyn argues against a tight physical interaction, but it does not necessarily mean that the two proteins do not interact at all. The physical interaction could be too transient in nature to be detected in a co-immunopercipitation experiment, but still able to induce a structural change in αSyn.

### RPS3A probably counteracts αSyn aggregation and toxicity through its extraribosomal chaperone function

When overexpressed in hepatocellular carcinoma cells, RPS3A was recently found to exert an extraribosomal chaperoning activity on HBx, preventing its aggregation into inclusions [18]. As we observe that RPS3A strongly counteracts the formation of αSyn–GFP inclusions in yeast cells, we suggest that the chaperoning function of RPS3A may also prevent the formation of toxic αSyn species, possibly by stabilization of a much less toxic plasma-membrane-bound species.

Because RPS3A can only partially complement the synthetic lethality of the *rps1a*Δ *rps1b*Δ strain and overexpression of yeast Rps1a or Rps1b does not suppress αSyn toxicity, it seems that the effect on αSyn is not related to the essential ribosomal function that is also exerted by the yeast homologues, but rather to an additional function that is specific for the mammalian counterpart. Lim et al. [18] found that the 50 N-terminal amino acids of RPS3A are essential for the interaction with and chaperone function on the HBx protein. In our yeast system, we observe that deletion of the 50 N-terminal amino acids abolishes the ability of RPS3A to suppress the αSyn-induced growth defect and inclusion formation, whereas the deletion of the 101 C-terminal amino acids did not affect suppression activity. When 151 or 214 amino acids are removed from the C-terminus, the suppression activity completely disappears. Hence, we have shown that the 50 N-terminal amino acids are essential for the chaperone function of RPS3A, but they are not sufficient. The fact that pRPS3AΔ164-264 has reduced suppression activity compared with full-length pRPS3A could be an indication that the N-terminus of RPS3A does play a role in promoting chaperone activity, or in the stability of the protein in yeast cells. Further evidence uncoupling the chaperone function of RPS3A from its ribosomal function comes from the fact that pRPS3AΔ164-263 does not support growth of *rps1a*Δ *rps1b*Δ cells, indicating that it cannot function as a ribosomal protein, whereas it does retain an effect on αSyn aggregation and toxicity.

**Figure 9:**
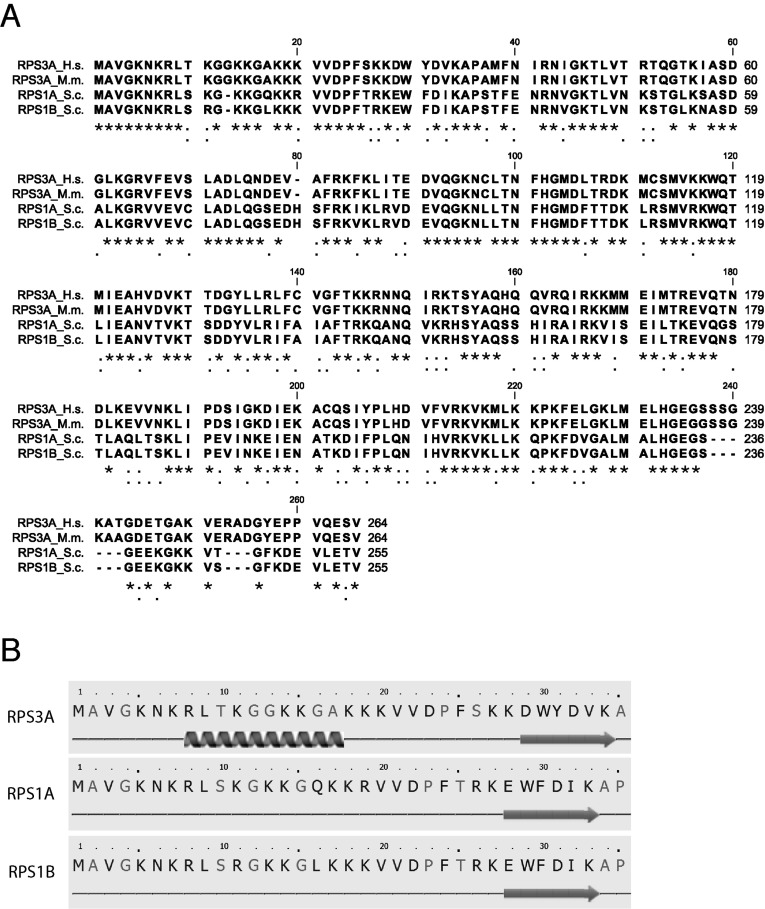
Differences in primary and predicted secondary structures of mouse or human RPS3A and yeast RPS1A or RPS1B (**A**) Sequence alignment of human (RPS3A_*H.s.*), mouse (RPS3A_*M.m.*) RPS3A, and yeast RPS1A and RPS1B (RPS1A_*S.c.* and RPS1A_*S.c.*). (**B**) Screen shots (only the N-terminal parts) of the result of secondary structure prediction by the ‘Protein Homology/AnalogY Recognition Engine’ Phyre2 [40] of mouse RPS3A, and yeast RPS1A and RPS1B. A helix under the sequence represents a predicted α-helix, an arrow represents a predicted β-sheet.

An alignment of mouse RPS3A with its yeast counterparts RPS1A and RPS1B shows 57% and 58% identities at the amino acid level respectively (Figure 9A). For completeness, the human RPS3A sequence (RPS3A_*H.s.*, NCBI accession number NP_000997) was also included in the alignment. It only differs from its mouse homologue in two amino acid positions, both located in the C-terminal part of the protein. The strongest sequence differences between the mammalian and the yeast proteins are in the 24 C-terminal amino acids of RPS3A, which show a very poor alignment, with large gaps. When the amino acid sequences were submitted to the secondary structure prediction program Phyre2 [40], the most prominent difference was in an α-helical region from Arg^8^ to Ala^17^ in mammalian RPS3A, which was not predicted in yeast RPS1A or RPS1B (Figure 9B). Since the N-terminal domain of RPS3A is essential for its chaperone function, we can speculate that this predicted α-helix may play an important role in establishing the chaperone activity.

### Conclusions

The present study has shown that a yeast selection system, employing a specific deletion strain highly sensitive to αSyn expression, can be used to directly isolate mammalian genes encoding proteins that affect αSyn-induced toxicity in yeast. One isolated suppressor (PSMA2) and one enhancer (the C-terminus of Spire homologue 1) did not act on αSyn directly, but mouse ribosomal subunit RPS3A clearly counteracted αSyn toxicity in all yeast backgrounds tested, suggesting a direct effect on the αSyn protein, possibly through its recently discovered extraribosomal chaperoning activity [18]. Overexpression of the yeast homologues of RPS3A did not result in suppression, suggesting a unique chaperone function for the mammalian protein. This illustrates the power of our screening set-up in identifying mammalian-specific effectors of αSyn phenotypes. The RPS3A clone strongly reduced the formation of αSyn–GFP inclusions in the *cog6*Δ2αSyn-GFP strain, indicating that it stabilizes an αSyn species with reduced toxicity and reduced propensity to form inclusions. The 50 N-terminal amino acids of RPS3A are essential for the effects on αSyn aggregation and toxicity, but this domain alone is not sufficient. Thus far, the functional interaction between RPS3A and αSyn has only been demonstrated in yeast cells. The next logical step will be to examine whether RPS3A can also influence αSyn-related phenotypes (such as inclusion formation and toxicity) in mammalian cells, in particular primary neuron cells.

Our discovery of a chaperone protein protecting against αSyn-induced toxicity suggests the possibility that αSyn is also toxic in normal human brain cells, but that its toxicity is suppressed by an active chaperone system, composed of RPS3A and possibly other chaperones. When neural cells age, this protective system may slowly degenerate, and/or under environmental stress, the system may become overloaded, causing αSyn to become toxic. Overexpression of αSyn or expression of specific mutant forms of αSyn, as in familial PD, may put a higher burden on the protective system so that the toxicity, and thus the onset of PD, would start at an earlier age.

## References

[1] Polymeropoulos M. H., Lavedan C., Leroy E., Ide S. E., Dehejia A., Dutra A., Pike B., Root H., Rubenstein J., Boyer R. (1997). Mutation in the α-synuclein gene identified in families with Parkinson's disease. Science.

[2] Spillantini M. G., Schmidt M. L., Lee V. M., Trojanowski J. Q., Jakes R., Goedert M. (1997). α-Synuclein in Lewy bodies. Nature.

[3] Goedert M. (2001). α-Synuclein and neurodegenerative diseases. Nat. Rev. Neurosci..

[4] Tan E. K., Skipper L. M. (2007). Pathogenic mutations in Parkinson disease. Hum. Mutat..

[5] Volles M. J., Lee S. J., Rochet J. C., Shtilerman M. D., Ding T. T., Kessler J. C., Lansbury P. T. (2001). Vesicle permeabilization by protofibrillar α-synuclein: implications for the pathogenesis and treatment of Parkinson's disease. Biochemistry.

[6] Gosavi N., Lee H. J., Lee J. S., Patel S., Lee S. J. (2002). Golgi fragmentation occurs in the cells with prefibrillar α-synuclein aggregates and precedes the formation of fibrillar inclusion. J. Biol. Chem..

[7] Thayanidhi N., Helm J. R., Nycz D. C., Bentley M., Liang Y., Hay J. C. (2010). α-Synuclein delays endoplasmic reticulum (ER)-to-Golgi transport in mammalian cells by antagonizing ER/Golgi SNAREs. Mol. Biol. Cell.

[8] Stefanis L., Larsen K. E., Rideout H. J., Sulzer D., Greene L. A. (2001). Expression of A53T mutant but not wild-type α-synuclein in PC12 cells induces alterations of the ubiquitin-dependent degradation system, loss of dopamine release, and autophagic cell death. J. Neurosci..

[9] Cuervo A. M., Stefanis L., Fredenburg R., Lansbury P. T., Sulzer D. (2004). Impaired degradation of mutant α-synuclein by chaperone-mediated autophagy. Science.

[10] Zhang N. Y., Tang Z., Liu C. W. (2008). α-Synuclein protofibrils inhibit 26 S proteasome-mediated protein degradation: understanding the cytotoxicity of protein protofibrils in neurodegenerative disease pathogenesis. J. Biol. Chem..

[11] Cookson M. R. (2009). α-Synuclein and neuronal cell death. Mol. Neurodegener..

[12] Willingham S., Outeiro T. F., DeVit M. J., Lindquist S. L., Muchowski P. J. (2003). Yeast genes that enhance the toxicity of a mutant huntingtin fragment or α-synuclein. Science.

[13] Franssens V., Boelen E., Anandhakumar J., Vanhelmont T., Buttner S., Winderickx J. (2009). Yeast unfolds the road map toward α-synuclein-induced cell death. Cell Death Differ..

[14] Auluck P. K., Caraveo G., Lindquist S. (2010). α-Synuclein: membrane interactions and toxicity in Parkinson's disease. Annu. Rev. Cell Dev. Biol..

[15] Cooper A. A., Gitler A. D., Cashikar A., Haynes C. M., Hill K. J., Bhullar B., Liu K., Xu K., Strathearn K. E., Liu F. (2006). α-Synuclein blocks ER-Golgi traffic and Rab1 rescues neuron loss in Parkinson's models. Science.

[16] Gitler A. D., Chesi A., Geddie M. L., Strathearn K. E., Hamamichi S., Hill K. J., Caldwell K. A., Caldwell G. A., Cooper A. A., Rochet J. C., Lindquist S. (2009). α-Synuclein is part of a diverse and highly conserved interaction network that includes PARK9 and manganese toxicity. Nat. Genet..

[17] Liang J., Clark-Dixon C., Wang S., Flower T. R., Williams-Hart T., Zweig R., Robinson L. C., Tatchell K., Witt S. N. (2008). Novel suppressors of α-synuclein toxicity identified using yeast. Hum. Mol. Genet..

[18] Lim K. H., Kim K. H., Choi S. I., Park E. S., Park S. H., Ryu K., Park Y. K., Kwon S. Y., Yang S. I., Lee H. C. (2011). RPS3a over-expressed in HBV-associated hepatocellular carcinoma enhances the HBx-induced NF-κB signaling via its novel chaperoning function. PLoS ONE.

[19] Bartels T., Choi J. G., Selkoe D. J. (2011). α-Synuclein occurs physiologically as a helically folded tetramer that resists aggregation. Nature.

[20] Van Mullem V., Wery M., De Bolle X., Vandenhaute J. (2003). Construction of a set of *Saccharomyces cerevisiae* vectors designed for recombinational cloning. Yeast.

[21] Versele M., Thorner J. (2004). Septin collar formation in budding yeast requires GTP binding and direct phosphorylation by the PAK, Cla4. J. Cell Biol..

[22] Guthrie C., Fink A. L. (1991). Guide to yeast genetics and molecular biology. Methods Enzymol..

[23] Sharma N., Brandis K. A., Herrera S. K., Johnson B. E., Vaidya T., Shrestha R., Debburman S. K. (2006). α-Synuclein budding yeast model: toxicity enhanced by impaired proteasome and oxidative stress. J. Mol. Neurosci..

[24] Outeiro T. F., Lindquist S. (2003). Yeast cells provide insight into α-synuclein biology and pathobiology. Science.

[25] Boeke J. D., Trueheart J., Natsoulis G., Fink G. R. (1987). 5-Fluoroorotic acid as a selective agent in yeast molecular genetics. Methods Enzymol..

[26] Zabrocki P., Pellens K., Vanhelmont T., Vandebroek T., Griffioen G., Wera S., Van Leuven F., Winderickx J. (2005). Characterization of α-synuclein aggregation and synergistic toxicity with protein tau in yeast. FEBS J..

[27] Griffioen G., Duhamel H., Van Damme N., Pellens K., Zabrocki P., Pannecouque C., van Leuven F., Winderickx J., Wera S. (2006). A yeast-based model of α-synucleinopathy identifies compounds with therapeutic potential. Biochim. Biophys. Acta.

[28] Volles M. J., Lansbury P. T. (2007). Relationships between the sequence of α-synuclein and its membrane affinity, fibrillization propensity, and yeast toxicity. J. Mol. Biol..

[29] Zabrocki P., Bastiaens I., Delay C., Bammens T., Ghillebert R., Pellens K., De Virgilio C., Van Leuven F., Winderickx J. (2008). Phosphorylation, lipid raft interaction and traffic of α-synuclein in a yeast model for Parkinson. Biochim. Biophys. Acta.

[30] Loh E., Hong W. (2004). The binary interacting network of the conserved oligomeric Golgi tethering complex. J. Biol. Chem..

[31] Suvorova E. S., Duden R., Lupashin V. V. (2002). The Sec34/Sec35p complex, a Ypt1p effector required for retrograde intra-Golgi trafficking, interacts with Golgi SNAREs and COPI vesicle coat proteins. J. Cell Biol..

[32] Laufman O., Hong W., Lev S. (2011). The COG complex interacts directly with syntaxin 6 and positively regulates endosome-to-TGN retrograde transport. J. Cell Biol..

[33] Kerkhoff E., Simpson J. C., Leberfinger C. B., Otto I. M., Doerks T., Bork P., Rapp U. R., Raabe T., Pepperkok R. (2001). The Spir actin organizers are involved in vesicle transport processes. Curr. Biol..

[34] Kim J. M., Lee K. H., Jeon Y. J., Oh J. H., Jeong S. Y., Song I. S., Lee D. S., Kim N. S. (2006). Identification of genes related to Parkinson**’**s disease using expressed sequence tags. DNA Res..

[35] Chin M. H., Qian W. J., Wang H., Petyuk V. A., Bloom J. S., Sforza D. M., Lacan G., Liu D., Khan A. H., Cantor R. M. (2008). Mitochondrial dysfunction, oxidative stress, and apoptosis revealed by proteomic and transcriptomic analyses of the striata in two mouse models of Parkinson's disease. J. Proteome Res..

[36] Grupe A., Li Y., Rowland C., Nowotny P., Hinrichs A. L., Smemo S., Kauwe J. S., Maxwell T. J., Cherny S., Doil L. (2006). A scan of chromosome 10 identifies a novel locus showing strong association with late-onset Alzheimer disease. Am. J. Hum. Genet..

[37] Minster R. L., DeKosky S. T., Kamboh M. I. (2006). Lack of association of two chromosome 10q24 SNPs with Alzheimer’s disease. Neurosci. Lett..

[38] Liang X., Schnetz-Boutaud N., Bartlett J., Allen M. J., Gwirtsman H., Schmechel D. E., Carney R. M., Gilbert J. R., Pericak-Vance M. A., Haines J. L. (2008). No association between SNP rs498055 on chromosome 10 and late-onset Alzheimer disease in multiple datasets. Ann. Hum. Genet..

[39] Bertram L., Hsiao M., Lange C., Blacker D., Tanzi R. E. (2006). Single-nucleotide polymorphism rs498055 on chromosome 10q24 is not associated with Alzheimer disease in two independent family samples. Am. J. Hum. Genet..

[40] Kelley L. A., Sternberg M. J. (2009). Protein structure prediction on the Web: a case study using the Phyre server. Nat. Protoc..

[41] Hu Y., Rolfs A., Bhullar B., Murthy T. V., Zhu C., Berger M. F., Camargo A. A., Kelley F., McCarron S., Jepson D. (2007). Approaching a complete repository of sequence-verified protein-encoding clones for *Saccharomyces cerevisiae*. Genome Res..

[42] Brachmann C. B., Davies A., Cost G. J., Caputo E., Li J., Hieter P., Boeke J. D. (1998). Designer deletion strains derived from *Saccharomyces cerevisiae* S288C: a useful set of strains and plasmids for PCR-mediated gene disruption and other applications. Yeast.

[43] Giaever G., Chu A. M., Ni L., Connelly C., Riles L., Veronneau S., Dow S., Lucau-Danila A., Anderson K., Andre B. (2002). Functional profiling of the *Saccharomyces cerevisiae* genome. Nature.

